# First Report on Yield and Chemical Composition of Essential Oil Extracted from *Myrcia eximia* DC (Myrtaceae) from the Brazilian Amazon

**DOI:** 10.3390/molecules25040783

**Published:** 2020-02-12

**Authors:** Oberdan Oliveira Ferreira, Jorddy Neves da Cruz, Celeste de Jesus Pereira Franco, Sebastião Gomes Silva, Wanessa Almeida da Costa, Mozaniel Santana de Oliveira, Eloisa Helena de Aguiar Andrade

**Affiliations:** 1 Program of Post-Graduation in biodiversity e biotecnology-Bionorte, Federal University of Para, Rua Augusto Corrêa S/N, Guamá, 66075-900 Belém, Brazil; oberdan@museu-goeldi.br; 2 Laboratório Adolpho Ducke Laboratory, Botany Coordination, Museu Paraense Emílio Goeldi, Av. Perimetral, 1900, Terra Firme, 66077-830 Belém, Brasil; jorddynevescruz@gmail.com; 3 Federal University of Para, Rua Augusto Corrêa S/N, Guamá, 66075-900 Belém, Brazil; celeste.franco@hotmail.com (C.d.J.P.F.); professebastiao@yahoo.com.br (S.G.S.); wanessa.almeida712@yahoo.com.br (W.A.d.C.)

**Keywords:** *Myrcia eximia* DC, essential oil, gas chromatography, (*E*)-caryophyllene

## Abstract

The essential oil (EO) of plants of the Myrtaceae family has diverse chemical composition and several applications. However, data on the oil yield, its composition, and its complete chemistry are still unavailable for some species belonging to this family, such as *Myrcia eximia* DC. In this study, the chemical compositions of the EOs of *Myrcia eximia* were evaluated by using gas chromatography (GC) alone and gas chromatography coupled with mass spectrometry (GC–MS). Samples for both evaluations were collected from the city of Magalhães Barata, State of Pará, Brazil, in 2017 and 2018. For the plant material collected in 2017, EO was obtained by hydrodistillation (HD) only, while, for the material collected in 2018, EO was obtained by hydrodistillation and steam distillation (SD), in order to evaluate the differences in chemical composition and mass yield of the EO. The yields of (*E*)-caryophyllene were 15.71% and 20.0% for the samples collected by HD in 2017 and 2018, respectively, while the yield was 15.0% for the sample collected by SD in 2018. Hexanal was found to be the major constituent in the EO obtained by HD, with yield of up to 26.09%. The oil yields reached 0.08% by using SD, and 0.01% and 0.36% for the samples collected in 2017 and 2018, respectively, using HD. The results of this study provide new information about the mass yield and chemical composition of *Myrcia eximia* DC, and they can add value and income to traditional populations, as well as facilitate the preservation of this species.

## 1. Introduction

Myrtaceae is one of the most important families of the Brazilian flora, and it has representatives of significant medicinal interest [1]. This family is composed of approximately 150 genera and 4630 species, especially distributed in the tropical and subtropical regions. It is widely dispersed in the Americas and in Australia, although it is found all over the world [2]. In Brazil, there are 23 genera and approximately 1034 species present throughout the country [3].

Recent studies on essential oils (EOs) isolated from plants of the Myrtaceae family showed that they have important properties, such as insecticidal, parasiticidal, antifungal, antibacterial, antimicrobial, and antioxidant activities [4]. This demonstrates the great importance of this family with respect to the discovery of new techniques, which can solve problems in various sectors, such as health, food, and even agricultural production.

The genus *Myrcia* DC is the most representative of the Myrtaceae family. In Brazil, it is represented by 23 genera and 974 species [5]. Many species of *Myrcia,* such as *M. Silvatica*, *M. punicifolia,* and *M. speciosa* are used in folk medicine, usually as infusions, for treating diabetes [6,7]. Others, such as *M. salicifolia* and *M. ovata* are used in the treatment of gastric diseases, diarrhea, cold sores, and mouth ulcers [6,8]. In addition, plants of the *Myrcia* species are sources of EOs with antibacterial, antinociceptive, and anti-inflammatory activities [9,10]. Sesquiterpenes and monoterpenes are the most frequently found components of their EOs [6].

Some studies conducted on the EOs of *Myrcia* species revealed their chemical diversity; they contained a wide range of chemicals, such as β-caryophyllene, germacrene B, δ-cadinene [11], α-pinene, α-terpineol [12], caryophyllene oxide, globulol, (*E*)-nerolidyl acetate, *ar*-curcumene, δ-cadinene, and spathulenol [13]. Studies on the species *Myrcia eximia* DC only focused on its anatomy and taxonomy. This species, popularly known in Brazil as “goiabinha”, is geographically distributed in the northeast, midwest, and southeast regions of Brazil [14,15]. Apart from a small report on the existence of β-caryophyllene [16], there is no literature available on the yield and chemical composition of its EO. In this context, herein, we aim to analyze the mass yield of EOs of *Myrcia eximia* DC collected in 2017 and 2018 from the city of Magalhães Barata, northeast Pará-Brazil, Eastern Amazon. We aim to garner new information for the dissemination of knowledge related to the chemical profile of the EOs of this species.

## 2. Results and Discussion

### 2.1. Yields

**Figure 1:**
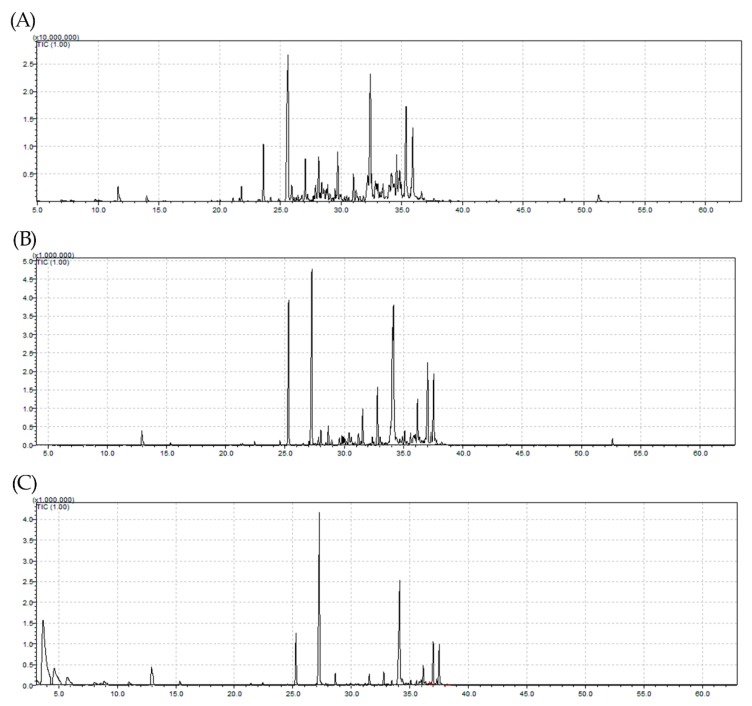
Ion chromatograms of *Myrcia eximia* DC essential oils (Eos) injected in GC/MS: (**A**) sample collected in 2017 by hydrodistillation (HD), (**B**) sample collected in 2018 by HD, and (**C**) sample collected in 2018 by steam distillation (SD). The *x-*axis represents the retention time, while the *y-*axis represents the relative concentration.

Moisture contents of 9.43% and 11.95% were obtained for leaf samples of *Myrcia eximia* DC collected in 2017 and 2018, respectively. This variation may be related to the collection period, because the sample with the highest moisture content was that collected in the rainy season. For samples obtained by hydrodistillation (HD), yields ranged from 0.01% to 0.36% (*w*/*w*), and the sample collected in the dry period of 2017 presented the highest mass yield of EO. This yield was 0.08% (*w*/*w*) for the same sample, when collected using steam distillation (SD). Figure 1 shows the chromatograms of the EO fractions collected in 2017 and 2018. Differences in the yields of EO fractions may be associated with the extraction technique employed. Other authors studying other plants compared the extraction methods of HD and steam distillation (SD) and reported that they can influence and induce differences in mass yields and chemical compositions at the end of the extraction process [17,18,19,20,21].

### 2.2. Chemical Composition of the EO

The samples were quantified and identified by using gas chromatography (GC) alone and gas chromatography combined with mass spectrometry (GC–MS). In total, 93 chemical compounds were identified, and they are listed in Table 1. To obtain the EO, two different techniques were used, HD and SD. For the plant material collected in 2017, EO was obtained only by HD, while, for the material collected in 2018, EO was obtained by HD and SD.

The main classes of compounds found in the sample collected in 2017 (dry season) were aldehydes (2.38%), hydrocarbon sesquiterpenes (36.21%), oxygenated sesquiterpenes (53.41%), and other compounds (0.27%), whereas, in the sample collected in 2018 (rainy season), there was large quantitative variation with respect to the classes of compounds obtained in the 2017 sample, i.e., 40.5% aldehydes, 23% hydrocarbon sesquiterpenes, and 30.5% oxygenated sesquiterpenes, as well as other compounds (0.2%), were identified. The different collection periods influenced the composition of this specimen of *M. eximia* because the aldehyde content increased, while the contents of hydrocarbon sesquiterpenes and oxygenated sesquiterpenes decreased.

All the EO samples presented qualitative and quantitative variations depending on the season of collection. In the dry period, (*E*)-caryophyllene (15.71%), caryophyllene oxide (10.25%), 14-hydroxy-9-*epi*-(*E*)-caryophyllene (7.02%), α-cadinol (5%), allohimachalol (3.49%), caryophylla-4(12), 8(13)-dien-5-α-ol (3.31%), and α-copaene (3.25%) were obtained as the main components. In the rainy period, hexanal (26.1%), (*E*)-caryophyllene (20.3%), caryophyllene oxide (16.3%), (2*E*)-hexenal (6.63%), α-copaene (4.84%), 14-hydroxy-9-*epi*-(*E*)-caryophyllene (4.63%), and nonanal (3.24%) were obtained as the major constituents. Therefore, EOs of the same species may vary qualitatively and quantitatively in composition, depending on the location, time of the day, climate, and season of the year [24,25,26].

The chemical constituents of the EO samples obtained by HD in 2017 and 2018 were different, and 96.93% and 98.84% of their components were identified, respectively.

By comparing the chemical constituents of these oils, the differences among the molecules can be identified. For instance, there are molecules that were identified only in the 2017 material obtained by HD, such as hexanal (26.09%) and (2*E*)-hexenal (6.63%). The difference in chemical compositions of these oils can have a direct impact on their biological activities, as well as their industrial and food applications.

From 2018 samples, hexanal (26.09%) and (2*E*)-hexenal (6.63%) could be obtained as major compounds by HD. These compounds were not found in the EO of the same plant sample obtained by SD, and they were not present in the oil extracted by HD of the material collected in 2017, either. Hexanal and (2*E*)-hexenal have antimicrobial activity against *Salmonella enteritidis, Escherichia coli, Listeria monocytogenes,* and *Aspergillus flavus* [27,28,29]; therefore, they can be used to extend the shelf life of minimally processed foods, such as apples, which are sold to customers, on a regular basis, ready to be consumed [29].

Nonanal was identified in the EO obtained by HD of the samples collected in 2017 and 2018, with its contents being 1.28% and 3.24%, respectively. This compound was also identified in the oil extracted by SD of the 2018 sample. The presence of this substance enhances the antimicrobial activity of the EO against bacterial and fungal pathogens. The minimum inhibitory concentration (MIC) and the minimum fungicidal concentration (MFC) against *Penicillium cyclopium* were investigated [30], and the results demonstrated that this volatile compound could alter the fungal hyphae morphology, leading to loss in cytoplasmic material and mycelial distortion. In addition, this substance caused severe changes in the permeability of fungal cell membranes. In this study, the authors obtained MIC = 0.3 mL/L and MFC = 0.4 mL/L, demonstrating that nonanal has suitable activity against *P. cyclopium* fungus. Zavala-Sánchez et al. [31] reported the antidiarrheal activity of nonanal, which showed significant inhibitory effects in mice with diarrhea induced by castor oil, magnesium sulfate, and arachidonic acid [31]. Nonanal was also used for alpha stimulation [32], and improvement of trap performance against *Aedes aegypti* [33].

α-Copaene was also identified, and its contents were 3.25% and 4.84% in the EO extracted from the samples collected in 2017 and 2018, respectively, by HD. The sample collected that same year was subjected to extraction by steam distillation, in which this compound was obtained in greater quantities (10.98%). This sesquiterpene has antioxidant and antigenotoxic activities [34]. There are also reports in the literature that host plants producing α-copaene are able to influence the mating of *Ceratitis capitate*, the male Mediterranean fruit fly [35].

Sesquiterpene (*E*)-caryophyllene was obtained as the major product in both the extraction methods used. When HD was used, (*E*)-caryophyllene contents were 15.71% and 20.27% in the 2017 and 2018 samples, respectively. When SD was used, (*E*)-caryophyllene content was 15.0%. (*E*)-Caryophyllene is a generally recognized as safe (GRAS) food cannabinoid, and its use is approved by the United States Food and Drug Administration (FDA). Its biological activities are widely reported in the literature, such as those against bacteria, [36], fungi [37], and viruses [38]. There are also reports of its anti-inflammatory [39], anticancer [40], analgesic [41], and antiphytoviral [42] activities. The analogs caryophyllene oxide and 14-hydroxy-9-*epi*-(*E*)-caryophyllene were also identified in the three extractions performed. 

Caryophyllene oxide was identified in the sample oil obtained by HD with contents of 10.25% and 16.31% in 2017 and 2018, respectively. When the 2018 sample was subjected to SD, caryophyllene oxide was obtained in greater quantity (22.16%). 14-Hydroxy-9-*epi*-(*E*)-caryophyllene was also obtained from the three extractions. Its contents were 7.02% and 4.63% in the oil obtained by HD of samples collected in 2017 and 2018, respectively, and 7.84% of the EO obtained by SD of the sample collected in 2018. There are several reports in the literature on plants in which these compounds are the major components of the EOs, and these were investigated in relation to their property of inducing programmed cell death in *Trypanosoma cruzi* [43] and antioxidant activity [44].

α-Cadinol constituted 5.00% and 0.10% of the total EOs obtained by HD of the 2017 and 2018 samples, respectively, while the oil obtained by SD contained 0.46% of this compound. The EO of plants containing α-cadinol are reported to have cytotoxic [45], anti-tyrosinase [46], and antimicrobial activities [47].

## 3. Materials and Methods

### 3.1. Plant Material

Leaf samples of *Myrcia eximia* DC were collected in two different periods from the city of Magalhães Barata, Pará, Brazil. The first sample was collected during the dry season (Amazonian summer), on 12 June 2017, at geographic coordinates of 00°47′51.6″ south (S) and 047°33′38.4″ west (W). The samples were identified by Dr. Antonio Elielson Sousa da Rocha and the incorporation of an *exsicata* in the Herbarium of Emílio Goeldi Museum, in the city of Belém, Pará, Brazil, under the registration number MG-231868. The second sample was collected in the rainy season (Amazonian winter), on 10 March 2018, at geographic coordinates of 00°47′54.2″ S and 047°33′5.56″ W with the incorporation of an *exsicata* in the Herbarium of Emílio Goeldi Museum, in the city of Belém, Pará, Brazil, under the registration number MG-237469.

### 3.2. Preparation and Characterization of the Raw Material

The leaf samples of *Myrcia eximia* DC were dried in an air-circulation oven for five days, at 35 °C, and then crushed in a knife mill (Tecnal, model TE-631/3, Piracicaba/SP, Brazil) at a speed of 2251 rpm for 10 min. The moisture content was analyzed by using a moisture analyzer (model IV2500, GEHAKA, Duquesa de Goiás, Real Parque, São Paulo, Brazil).

### 3.3. Hydrodistillation

Hydrodistillation was performed on a Clevenger-type apparatus [48,49], using 176.29 g of the plant material collected in 2017 and 2018. The extraction period was 10,800 s with a temperature of 100 °C. After extraction, anhydrous sodium sulfate (Na_2_SO_4_) was added, and the EO was centrifuged to eliminate moisture. The mass yield of the EO was calculated on dry basis (db), by relating the oil mass obtained by HD and the dry mass used in the extraction process, according to Equation (1).

(1) $$\%\;yield\;oil\left(\frac{w}{w}\right)db=\;\;\frac{m_{oil}}{m_{sample}-\;\left(humidity\;\left(\%\right)\right)}\times \;100.$$

### 3.4. Steam Distillation

For extraction by SD [50], 100 g of MG-231868 (vegetable material collected in 2018) was used. The extraction time was 10,000 s, and the yield was calculated according to Equation (1).

### 3.5. Analysis of Volatile Compounds

The chemical composition of the EOs was evaluated according to a reported methodology [51], by using gas chromatography/mass spectrometry (Shimadzu, QP-2010 plus system, (City Kyoto, Japan), under the following conditions: silica capillary column Rtx-5MS (30 m × 0.25 mm, film thickness = 0.25 μm), program temperature of 60–240 °C (3 °C/min), injector temperature of 250 °C, helium as drag gas (linear velocity of 32 cm/s, measured at 100 °C), and splitless injection (1 μL of a 2:1000 hexane solution). Ionization was obtained by the electronic impact technique at 70 eV; the temperature of the ion source and other parts was 200 °C. The volatile compounds were quantified by gas chromatography using a flame ionization detector (FID) (Shimadzu, QP 2010 system), under the same conditions as GC/MS, except that nitrogen was used as the drag gas. The retention index was calculated for all the volatile constituents using a homologous series of *n*-alkanes (C8–C20). They were identified by comparison of their mass spectra and retention indices to those reported in the literature [22,52].

## 4. Conclusions

High concentrations of oxygenated sesquiterpenes were found in the EOs of *Myrcia eximia* DC specimens collected in 2017 and 2018, among which (*E*)-caryophyllene gained prominence in the chemical composition of both specimens. Aldehydes were responsible for the characterization of the 2018 sample (HD) oils, with emphasis on hexanal. Notably, hydrocarbon sesquiterpenes are commonly found in the chemical composition of EOs of the genus *Myrcia*, such as (*E*)-caryophyllene. The results of this study of *M. eximia* can contribute to dissemination of knowledge regarding the chemical composition of this species, which is almost incipient in the literature. As noted, important molecules were identified in the *Myrcia eximia* DC essential oil, which shows that this species can be a natural source of chemically active substances for a wide range of industrial applications.

## Figures and Tables

**Table 1:** Chemical composition of essential oils extracted from leaves of *Myrcia eximia* DC, at different periods, by hydrodistillation (HD) and steam distillation (SD).

RI (C)	RI (L)	Constituents	2017	2018	
			HD	HD	SD
798	801	Hexanal		26.1	
845	846	(2*E*)-Hexenal		6.63	
901	901	Heptanal		1.78	
1003	998	Octanal		0.59	
1009	1005	(2*E*,4*E*)-Heptadienal		0.24	
1054	1049	(2*E*)-Octen-1-al	0.2	0.69	
1062	1060	(2*E*)-Octen-1-ol	0.05		
1104	1100	Nonanal	1.28	3.24	1.42
1158	1157	(2*E*)-Nonen-1-al	0.41	0.53	0.2
1190	1186	α-Terpineol	0.05		
1194	1190	Methyl salicylate	0.05		
1294	1292	(2*E*,4*Z*)-Decadienal	0.12	0.18	0.1
1289	1299	10-Undecenal	0.06		0.04
1318	1315	(2*E*,4*E*)-Decadienal	0.2	0.23	0.22
1333	1335	δ-Elemene	0.96		
1345	1345	α-Cubebene	0.05		
1362	1357	Undec-(2*E*)-enal	0.11	0.1	0.27
1369	1373	α-Ylangene	0.16		
1374	1374	α-Copaene	3.25	4.84	10.98
1387	1389	β-Elemene	0.24		
1402	1400	β-Longipinene	0.19		
1404	1403	Eugenol methyl			
1418	1415	(2*E*,4*E*)-Undecadienal		0.14	0.25
1420	1417	(*E*)-Caryophyllene	15.71	20.3	15
1426	1419	β-Ylangene			0.19
1429	1428	(*E*)-α-Ionone			0.12
1431	1432	*trans*-α-Bergamotene	0.25	0.15	0.49
1435	1434	ϒ-Elemene	1.08		0.07
1438	1439	Aromadendrene	0.25		0.97
1441	1440	(*Z*)-β-Farnesene		0.08	
1442	1442	Guaia-6,9-diene	0.31		
1445	1447	Isogermacrene D	0.65		
1453	1451	*trans*-Muurola-3,5-diene	0.13		
1454	1452	α-Humulene	2.41	1.03	1.37
1455	1453	Geranyl acetone		0.02	0.19
1457	1458	Alloaromadendrene	0.4		0.45
1465	1464	9-*epi*-(*E*)-Caryophyllene	0.04		
1466	1465	Thujopsadiene	0.05		
1470	1471	Dauca-5,8-diene	0.35		
1475	1478	ϒ-Muurolene	1.1		
1480	1479	α-Curcumene			0.65
1484	1483	α-Amorphene	0.42		
1486	1483*	*trans*-β-Bergamotene		0.13	0.51
1487	1484	Germacrene D	2.93	0.08	0.58
1490	1489	β-Selinene			0.46
1491	1489	*cis*-Eudesma-6,11-diene		0.1	
1492	1492	*cis*-β-Guaiene	1.21		
1495	1496	Valencene			0.3
1498	1496	Viridiflorene	1.03		1.29
1499	1500	α-Muurolene	0.89		0.8
1508	1505	Premnaspirodiene			0.09
1510	1505	β-Bisabolene			0.29
1511	1509	Guaia-1(10),11-diene	0.8		
1512	1509	α-Bulnesene	0.16		
1513	1511	δ-Amorphene	0.64	0.05	
1514	1513	ϒ-Cadinene	0.63		
1515	1514	β-Curcumene			0.04
1516	1514	Cubebol		0.21	1.03
1521	1518	Dodecadienal (2*E*,4*E*)		0.09	0.31
1522	1522	δ-Cadinene	2.75	1.1	2.61
1523	1528	Zonarene	0.44		
1528	1531	(*Z*)-Nerolidol	0.35		
1531	1533	*trans*-Cadina-1,4-diene	0.21		
1537	1537	α-Cadinene	0.29		
1542	1544	α-Calacorene	0.23		0.58
1546	1547	Italicene epoxide	1.57	1.22	4.37
1556	1556	*trans*-Dauca-4(11),7-diene	0.1		
1563	1561	(*E*)-Nerolidol		0.09	0.22
1564	1562	*epi*-Longipinanol	0.86	0.15	0.71
1567	1567	Palustrol	0.31		
1572	1570	Dendrolasin			0.21
1575	1571	Caryolan-8-ol			0.22
1576	1577	Spathulenol	2.67		1.5
1581	1582	Caryophyllene oxide	10.25	16.3	22.16
1582	1484	Germacrene B	0.56		
1590	1586	Thujopsan-2α-ol			0.69
1592	1589	Allohedycayol			0.53
1593	1590	Globulol	1.78		
1595	1592	Viridiflorol	0.72	0.16	
1596	1595	Cubeban-11-ol	1.7		
1597	1596	Fokienol	0.98		
1605	1600	Rosifoliol	0.5		0.2
1607	1608	Humulene epoxide II	1.05	0.36	1.07
1615	1618	1,10-di-*epi*-Cubenol	0.74		
1620	1618	Junenol	1.18		
1625	1627	1-*epi*-Cubenol	1.85	0.48	0.52
1627	1629	Eremoligenol	0.58		0.2
1632	1635	*cis*-Cadin-4-en-7-ol	1.18		
1641	1638	*epi*-α-Cadinlol	1.17		
1642	1639	Caryophylla-4(12),8(13)-dien-5-α-ol	3.31	1.69	3.35
1643	1639	Alloromadendrene epoxide	1.13	0.49	1.57
1644	1640	*epi*-α-Muurolol	1.71	0.39	
1645	1644	α-Muurolol	1.21		
1651	1645	Cubenol		0.11	
1656	1649	β-Eudesmol		0.22	0.28
1359	1652	Himachalol		0.15	0.4
1656	1652	α-Cadinol	5.0	0.1	0.46
1660	1658	Selin-11-en-4α-ol			0.58
1662	1661	Allohimachalol	3.49		
1670	1668	14-Hydroxy-9-*epi*-(*E*)-caryophyllene	7.02	4.63	7.84
1688	1685	Germacra-4(15),5,10(14)-trien-1α-ol	0.72	3.7	
1696	1700	Eudesm-7(11)-en-4-ol	0.21		
1699	1706	14-Hydroxy-4,5-dihydro-caryophyllene			0.17
1711	1713	14-Hydroxy-α-humulene	0.09		
1712	1714	Nootkatol	0.08		
1734	1740	Mint sulfide	0.05		
1840	1841	Phytone	0.05		0.08
1944	1942	Phytol	0.02		0.43
		Aldehydes	2.38	40.5	2.81
		Hydrocarbon sesquiterpenes	36.21	23	26.74
		Oxygenated sesquiterpenes	53.41	30.5	53.89
		Others	0.27	0.02	0.85
		Total	96.93	98.8	95.27

RI (C): Retention index calculated from a series of *n*-alkanes (C8–C40) in column DB-5MS. RI (L): Retention index found in the literature—Adams [22], Mondello* [23].

## Supplementary Files

Supplementary File 1

## References

[1] Carneiro N.S., Alves C.C.F., Alves J.M., Egea M.B., Martins C.H.G., Silva T.S., Bretanha L.C., Balleste M.P., Micke G.A., Silveira E.V. (2017). Chemical composition, antioxidant and antibacterial activities of essential oils from leaves and flowers of Eugenia klotzschiana Berg (Myrtaceae). An. Acad. Bras. Cienc..

[2] Dluzniewski F.D.S., Vettorato J.G., Ghellar Müller N.T. (2018). Abordagem etnobotânica de Myrtaceae no município de Sete de Setembro, Rio Grande do Sul, Brasil. Rev. Interdiscip. Em Ciências Da Saúde E Biológicas – Ricsb.

[3] Santos C.d., Galaverna R.S., Angolini C.F.F., Nunes V.V.A., de Almeida L.F.R., Ruiz A.L.T.G., de Carvalho J.E., Duarte R.M.T., Duarte M.C.T., Eberlin M.N. (2018). Antioxidative, antiproliferative and antimicrobial activities of phenolic compounds from three myrcia species. Molecules.

[4] da Silva V.P., Alves C.C.F., Miranda M.L.D., Bretanha L.C., Balleste M.P., Micke G.A., Silveira E.V., Martins C.H.G., Ambrosio M.A.L.V., de Souza Silva T. (2018). Chemical composition and in vitro leishmanicidal, antibacterial and cytotoxic activities of essential oils of the Myrtaceae family occurring in the Cerrado biome. Ind. Crops Prod..

[5] Stadnik A., Oliveira M.I.U., de Roque N. (2016). Floristic survey of Myrtaceae in Jacobina municipality, Chapada Diamantina, Bahia State, Brazil. Hoehnea.

[6] Cascaes M.M., Guilhon G.M.S.P., de Aguiar Andrade E.H., das Graças Bichara Zoghbi M., da Silva Santos L. (2015). Constituents and pharmacological activities of Myrcia (Myrtaceae): A review of an aromatic and medicinal group of plants. Int. J. Mol. Sci..

[7] Scalvenzi L., Grandini A., Spagnoletti A., Tacchini M., Neill D., Ballesteros J.L., Sacchetti G., Guerrini A. (2017). Myrcia splendens (Sw.) DC. (syn. M. fallax (Rich.) DC.) (myrtaceae) essential oil from amazonian Ecuador: A chemical characterization and bioactivity profile. Molecules.

[8] Cândido C.S., Portella C.S.A., Laranjeira B.J., da Silva S.S., Arriaga A.M.C., Santiago G.M.P., Gomes G.A., Almeida P.C., Carvalho C.B.M. (2010). Effects of Myrcia ovata Cambess. essential oil on planktonic growth of gastrointestinal microorganisms and biofilm formation of Enterococcus faecalis. Brazilian J. Microbiol..

[9] Andrade G.S., Guimarães A.G., Santana M.T., Siqueira R.S., Passos L.O., Machado S.M.F., Ribeiro A.D.S., Sobral M., Almeida J.R.G.S., Quintans-Júnior L.J. (2011). Phytochemical screening, antinociceptive and anti-inflammatory effects of the essential oil of Myrcia pubiflora in mice. Brazilian J. Pharmacogn..

[10] Jiménez D., Araque M., Rojas L., Cordero A., Briceño B. (2012). Componentes volátiles y actividad antibacteriana del vástago de Myrcia splendens (Sw.) DC. Rev. la Fac. Farm..

[11] Do Silva A.N., Uetanabaro A.P.T., Lucchese A.M. (2013). Chemical composition and antibacterial activity of essential oils from Myrcia alagoensis (Myrtaceae). Nat. Prod. Commun..

[12] De Cerqueira M.D., Souza-Neta L.C., Passos M.D.G.V.M., Lima E.D.O., Roque N.F., Martins D., Guedes M.L.S., Cruz F.G. (2007). Seasonal variation and antimicrobial activity of Myrcia myrtifolia essential oils. J. Braz. Chem. Soc..

[13] Limberger R.P., Sobral M., Henriques A.T., Menut C., Bessière J.M. (2004). Óleos voláteis de espécies de Myrcia nativas do Rio Grande do Sul. Quim. Nova.

[14] Amaral D.D., Viera I.C.G., Salomão R.P., de Almeida S.S., Jardim M.A.G. (2009). Checklist da Flora Arbórea de Remanescentes Florestais da Região Metropolitana de Belém, Pará, Brasil. Bol. do Mus. Para. Emilio Goeldi Ciências Nat..

[15] Grandtner M.M., Chevrette J. (2013). Dictionary of Trees.

[16] Maia O.G.S., Andrade L.H.A. (2009). Database of the amazon aromatic plants and their essential oils. Quim. Nova..

[17] Naz S., Hanif M.A., Bhatti H.N., Ansari T.M. (2017). Impact of Supercritical Fluid Extraction and Traditional Distillation on the Isolation of Aromatic Compounds from Cannabis indica and Cannabis sativa. J. Essent. Oil Bear. Plants.

[18] Abd El-Gaber A.S., El Gendy A.N.G., Elkhateeb A., Saleh I.A., El-Seedi H.R. (2018). Microwave Extraction of Essential Oil from Anastatica hierochuntica (L): Comparison with Conventional Hydro-Distillation and Steam Distillation. J. Essent. Oil Bear. Plants.

[19] Khanavi M., Hadjiakhoondi A., Amin G., Amanzadeh Y., Rustaiyan A., Shafiee A. (2004). Comparison of the Volatile Composition of Stachys persica Gmel. and Stachys byzantina C. Koch. Oils Obtained by Hydrodistillation and Steam Distillation. Zeitschrift für Naturforsch. C.

[20] Conde-Hernández L.A., Espinosa-Victoria J.R., Trejo A., Guerrero-Beltrán J.Á. (2017). CO_2_ -supercritical extraction, hydrodistillation and steam distillation of essential oil of rosemary ( Rosmarinus officinalis ). J. Food Eng..

[21] Périno-Issartier S., Ginies C., Cravotto G., Chemat F. (2013). A comparison of essential oils obtained from lavandin via different extraction processes: Ultrasound, microwave, turbohydrodistillation, steam and hydrodistillation. J. Chromatogr. A.

[22] Adams R.P. (2007). Identification of Essential Oil Components by Gas Chromatography/Mass Spectroscopy.

[23] Mondello L. (2011). FFNSC 2: Flavors and Fragrances of Natural and Synthetic Compounds, Mass Spectral Database.

[24] Silva S.G., Figueiredo P.L.B., Nascimento L.D., da Costa W.A., Maia J.G.S., Andrade E.H.A. (2018). Planting and seasonal and circadian evaluation of a thymol-type oil from Lippia thymoides Mart. & Schauer. Chem. Cent. J..

[25] Ribeiro A.F., Andrade E.H.A., Salimena F.R.G., Maia J.G.S. (2014). Circadian and seasonal study of the cinnamate chemotype from Lippia origanoides Kunth. Biochem. Syst. Ecol..

[26] Bezerra F.W.F., de Oliveira M.S., Bezerra P.N., Cunha V.M.B., Silva M.P., da Costa W.A., Pinto R.H.H., Cordeiro R.M., da Cruz J.N., Chaves Neto A.M.J. (2020). Extraction of bioactive compounds. Green Sustainable Process for Chemical and Environmental Engineering and Science.

[27] Gardini F., Lanciotti R., Guerzoni M.E. (2001). Effect of trans-2-hexenal on the growth of Aspergillus flavus in relation to its concentration, temperature and water activity. Lett. Appl. Microbiol..

[28] Trombetta D., Saija A., Bisignano G., Arena S., Caruso S., Mazzanti G., Uccella N., Castelli F. (2002). Study on the mechanisms of the antibacterial action of some plant alpha,beta-unsaturated aldehydes. Lett. Appl. Microbiol..

[29] Lanciotti R., Belletti N., Patrignani F., Gianotti A., Gardini F., Guerzoni M.E. (2003). Application of Hexanal, (*E*)-2-Hexenal, and Hexyl Acetate To Improve the Safety of Fresh-Sliced Apples. J. Agric. Food Chem..

[30] Zhang J., Sun H., Chen S., Zeng L., Wang T. (2017). Anti-fungal activity, mechanism studies on α-Phellandrene and Nonanal against Penicillium cyclopium. Bot. Stud..

[31] Zavala-Sánchez M.A., Pérez-Gutiérrez S., Pérez-González C., Sánchez-Saldivar D., Arias-García L. (2002). Antidiarrhoeal activity of nonanal, an aldehyde isolated from Artemisia ludoviciana. Pharm. Biol..

[32] Kim M., Sowndhararajan K., Choi H.J., Park S.J., Kim S. (2019). Olfactory Stimulation Effect of Aldehydes, Nonanal, and Decanal on the Human Electroencephalographic Activity, According to Nostril Variation. Biomedicines.

[33] Barbosa R.M.R., Furtado A., Regis L., Leal W.S. (2010). Evaluation of an oviposition-stimulating kairomone for the yellow fever mosquito, Aedes aegypti, in Recife, Brazil. J. Vector Ecol..

[34] Turkez H., Togar B., Tatar A., Geyıkoglu F., Hacımuftuoglu A. (2014). Cytotoxic and cytogenetic effects of α-copaene on rat neuron and N2a neuroblastoma cell lines. Biologia.

[35] Shelly T.E. (2006). Exposure to α-Copaene and α-Copaene-Containing Oils Enhances Mating Success of Male Mediterranean Fruit Flies (Diptera: Tephritidae). Ann. Entomol. Soc. Am..

[36] Pieri F.A., de Castro Souza M.C., Vermelho L.L.R., Vermelho M.L.R., Perciano P.G., Vargas F.S., Borges A.P.B., da Veiga-Junior V.F., Moreira M.A.S. (2016). Use of β-caryophyllene to combat bacterial dental plaque formation in dogs. BMC Vet. Res..

[37] Cipriano M., Neta S., Vittorazzi C., Guimarães A.C., Damasceno J., Martins L., Fronza M., Coutinho Endringer D., Scherer R., Guimar A.C. (2017). Pharmaceutical Biology Effects of β-caryophyllene and Murraya paniculata essential oil in the murine hepatoma cells and in the bacteria and fungi 24-h time-kill curve studies. Pharm. Biol..

[38] Venturi C.R., Danielli L.J., Klein F., Apel M.A., Montanha J.A., Bordignon S.A.L., Roehe P.M., Fuentefria A.M., Henriques A.T. (2015). Pharmaceutical Biology Chemical analysis and in vitro antiviral and antifungal activities of essential oils from Glechon spathulata and Glechon marifolia Chemical analysis and in vitro antiviral and antifungal activities of essential oils from Glechon spa. John M. Pezzuto Pharm. Biol..

[39] Brito L.F., Oliveira H.B.M., Neves Selis N., Souza C.L.S., Júnior M.N.S., Souza E.P., Silva L.S.C.d., Souza Nascimento F., Amorim A.T., Campos G.B. (2019). Anti-inflammatory activity of *β*-caryophyllene combined with docosahexaenoic acid in a model of sepsis induced by *Staphylococcus aureus* in mice. J. Sci. Food Agric..

[40] Dahham S.S., Tabana Y.M., Iqbal M.A., Ahamed M.B.K., Ezzat M.O., Majid A.S.A., Majid A.M.S.A. (2015). The anticancer, antioxidant and antimicrobial properties of the sesquiterpene β-caryophyllene from the essential oil of Aquilaria crassna. Molecules.

[41] Fidyt K., Fiedorowicz A., Strządała L., Szumny A. (2016). *β* -caryophyllene and *β* -caryophyllene oxide-natural compounds of anticancer and analgesic properties. Cancer Med..

[42] Vuko E., Rusak G., Dunkic V., Kremer D., Kosalec I., Rada B., Bezic N. (2019). Inhibition of satellite RNA associated cucumber mosaic virus infection by essential oil of micromeria croatica (pers.) schott. Molecules.

[43] Moreno É.M., Leal S.M., Stashenko E.E., García L.T. (2018). Induction of programmed cell death in Trypanosoma cruzi by Lippia alba essential oils and their major and synergistic terpenes (citral, limonene and caryophyllene oxide). BMC Complement. Altern. Med..

[44] de Souza Araújo C., Paula de Oliveira A., Nascimento Lima R., Barreto Alves P., Coimbra Diniz T., Roberto Guedes da Silva Almeida J. (2015). Chemical constituents and antioxidant activity of the essential oil from leaves of Annona vepretorum Mart. (Annonaceae). Pharmacogn. Mag..

[45] Guerrini A., Sacchetti G., Grandini A., Spagnoletti A., Asanza M., Scalvenzi L. (2016). Cytotoxic Effect and TLC Bioautography-Guided Approach to Detect Health Properties of Amazonian Hedyosmum sprucei Essential Oil. Evidence-based Complement. Altern. Med..

[46] Zardi-Bergaoui A., Jelizi S., Flamini G., Ascrizzi R., Ben Jannet H. (2018). Comparative study of the chemical composition and bioactivities of essential oils of fresh and dry seeds from Myoporum insulare R. Br. Ind. Crops Prod..

[47] Ali N., Chhetri B., Dosoky N., Shari K., Al-Fahad A., Wessjohann L., Setzer W. (2017). Antimicrobial, Antioxidant, and Cytotoxic Activities of Ocimum forskolei and Teucrium yemense (Lamiaceae) Essential Oils. Medicines.

[48] de Oliveira M.S., da Cruz J.N., Gomes Silva S., da Costa W.A., de Sousa S.H.B., Bezerra F.W.F., Teixeira E., da Silva N.J.N., de Aguiar Andrade E.H., de Jesus Chaves Neto A.M. (2019). Phytochemical profile, antioxidant activity, inhibition of acetylcholinesterase and interaction mechanism of the major components of the Piper divaricatum essential oil obtained by supercritical CO. J. Supercrit. Fluids.

[49] Silva S.G., da Costa R.A., de Oliveira M.S., da Cruz J.N., Figueiredo P.L.B., Brasil D.d.S.B., Nascimento L.D., Chaves Neto A.M.d.J., de Carvalho Junior R.N., Andrade E.H.d.A. (2019). Chemical profile of Lippia thymoides, evaluation of the acetylcholinesterase inhibitory activity of its essential oil, and molecular docking and molecular dynamics simulations. PLoS ONE.

[50] Lopes N.P., Kato M.J., de Aguiar Andrade E.H., Soares Maia J.G., Yoshida M. (1997). Circadian and seasonal variation in the essential oil from Virola surinamensis leaves. Phytochemistry.

[51] Gurgel E.S.C., de Oliveira M.S., Souza M.C., da Silva S.G., de Mendonça M.S., Souza Filho A.P.d.S. (2019). Chemical compositions and herbicidal (phytotoxic) activity of essential oils of three Copaifera species (Leguminosae-Caesalpinoideae) from Amazon-Brazil. Ind. Crops Prod..

[52] Stein S., Mirokhin D., Tchekhovskoi D., Mallard G., Mikaia A., Zaikin V., Sparkmanm D. (2011). The NIST Mass Spectral Search Program for the Nist/Epa/Nih Mass Spectra Library.

