# Supplementary material for: First Report on Yield and Chemical Composition of Essential Oil Extracted from Myrcia eximia DC (Myrtaceae) from the Brazilian Amazon
Source: Molecules. 2020 Feb 12;25(4):783. doi: 10.3390/molecules25040783 (PMC7070909; doi:10.3390/molecules25040783)
Supplement: Supplementary File 1 [file molecules-25-00783-s001.pdf]

Supplementary Material S1. Mass spectrum of identified compounds in *Myrcia eximia* essential oil.

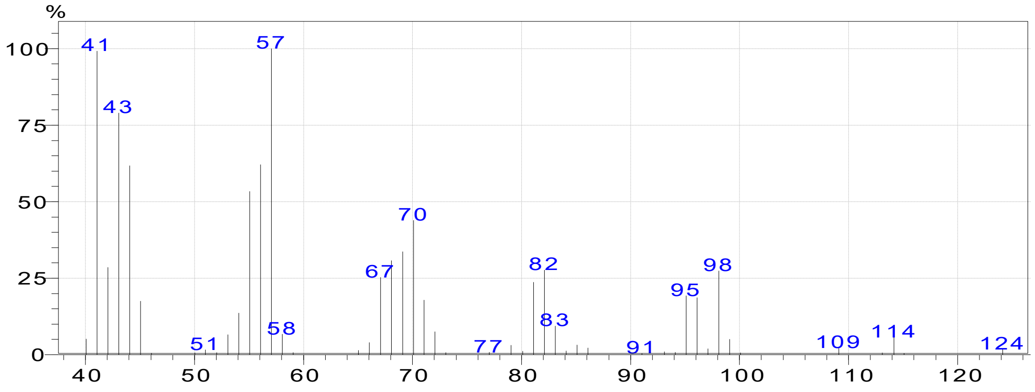

Figura 1. Mass spectrum of n-nonanal

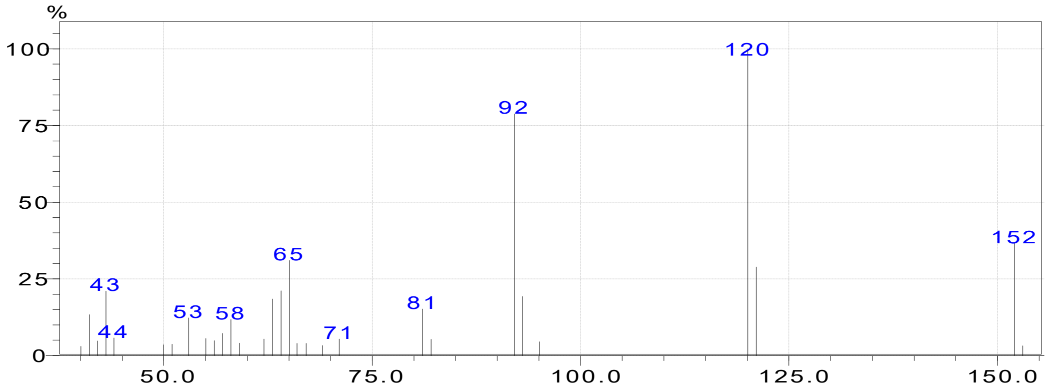

Figura 2. Mass spectrum of Salicylate <methyl->

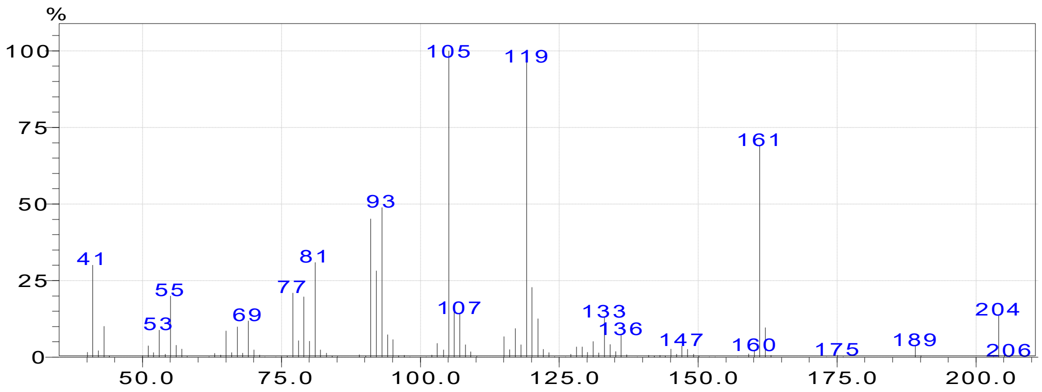

Figura 3. Mass spectrum of αlfa-copaene

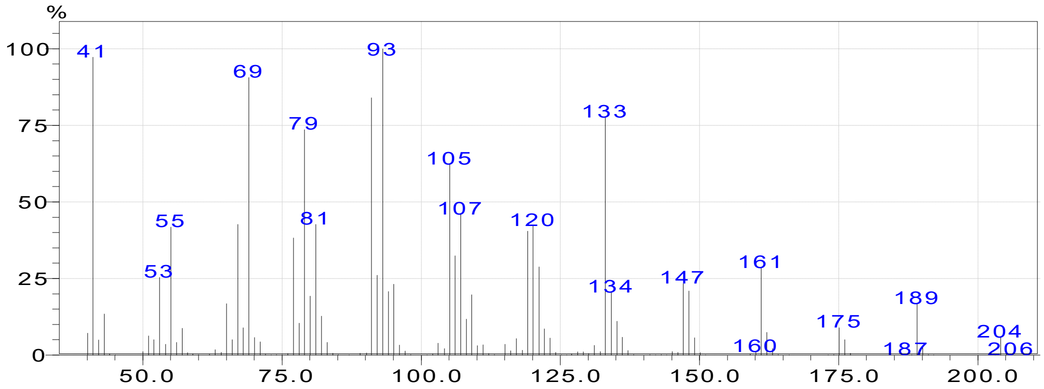

Figura 4. Mass spectrum of *E-Caryophyllene*

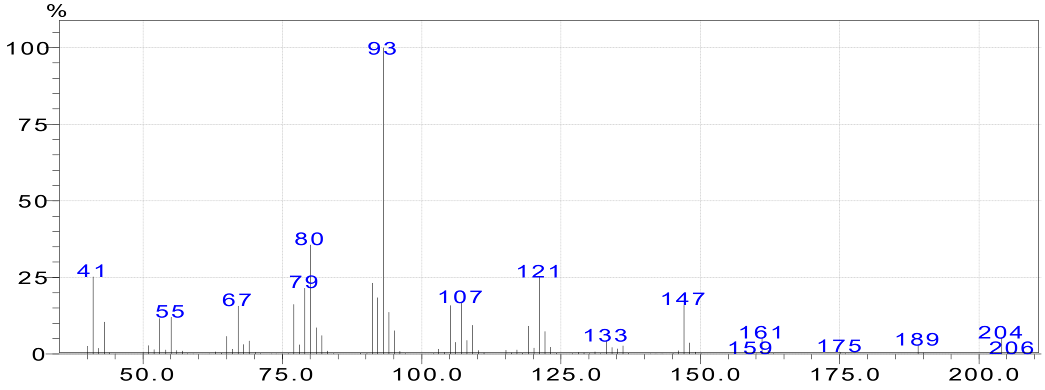

Figura 5. Mass spectrum of alfa-humuleno

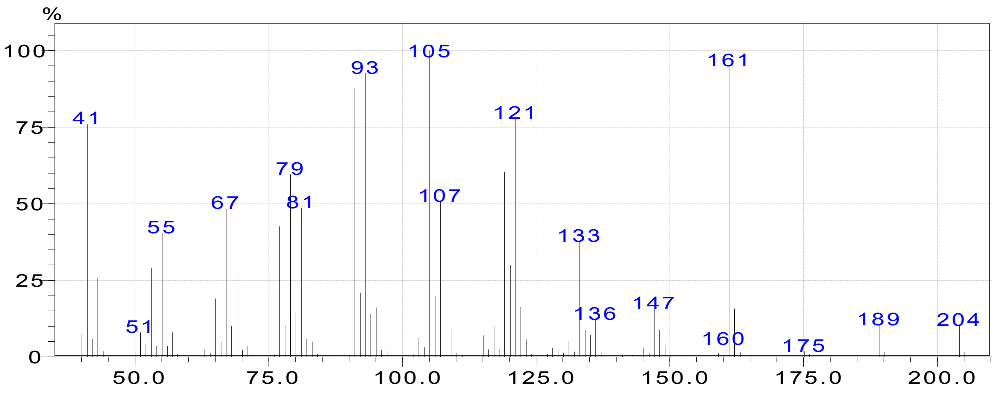

Figura 6. Mass spectrum of Elemene <gamma-

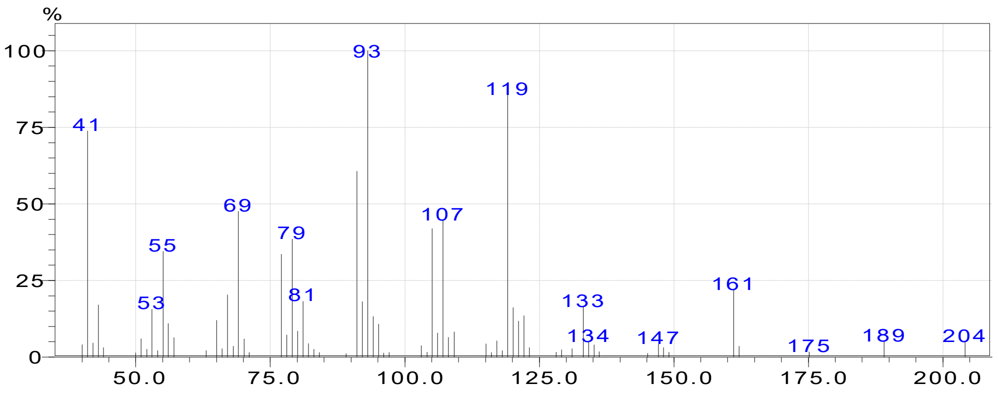

Figura 7. Mass spectrum of trans-.alpha.-Bergamotene

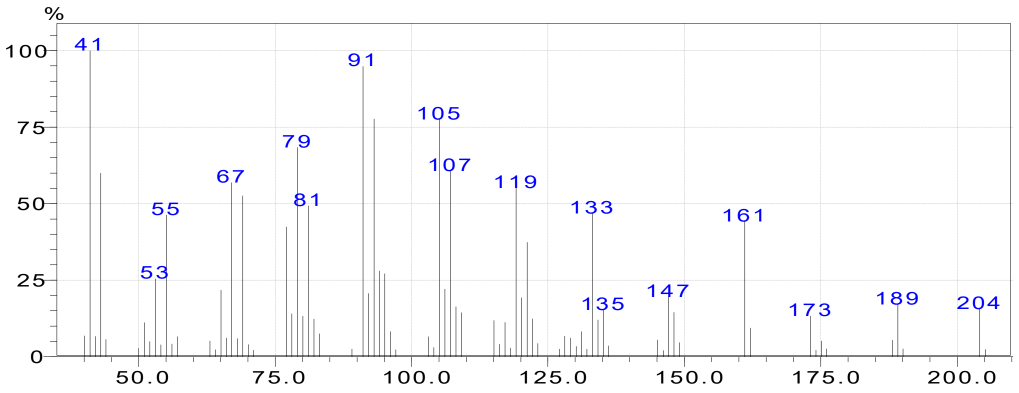

Figura 8. Mass spectrum of *Aromadendrene*

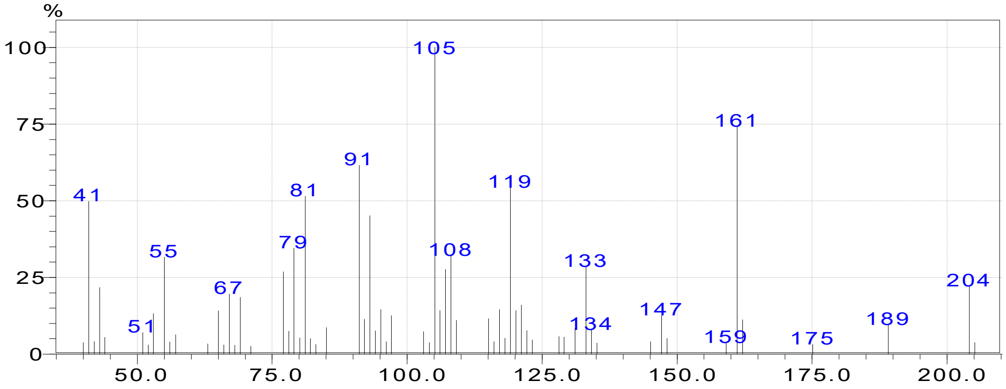

Figura 9. Mass spectrum of Guaiadiene<6,9->

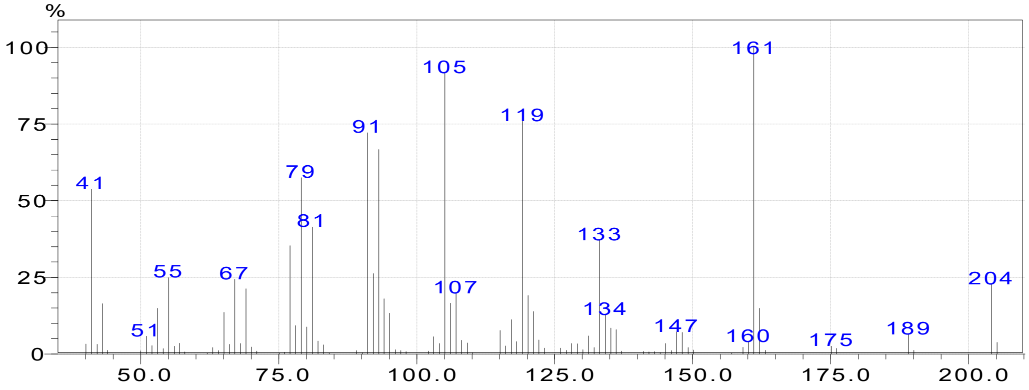

Figura 10. Mass spectrum of gama-muuroleno

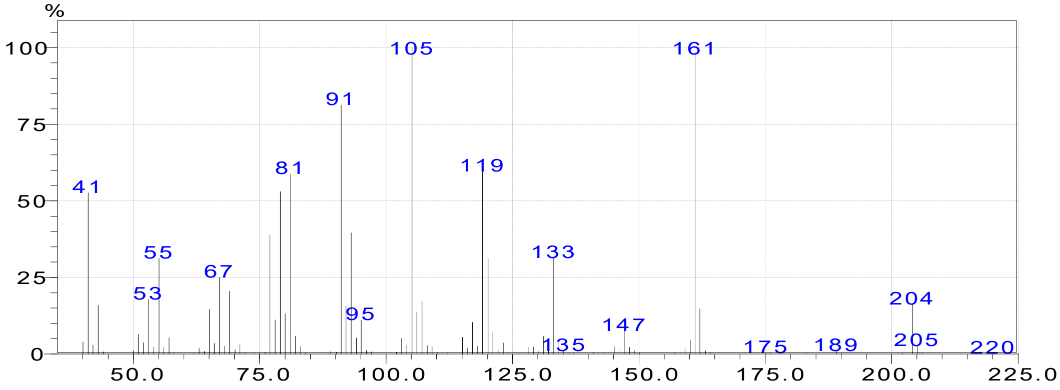

Figura 11. Mass spectrum of germacreno D

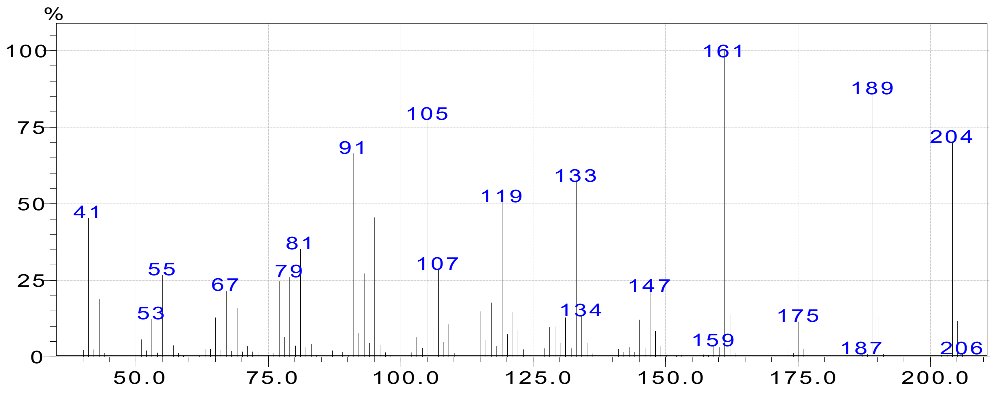

Figura 12. Mass spectrum of Guaiene<cis-beta->

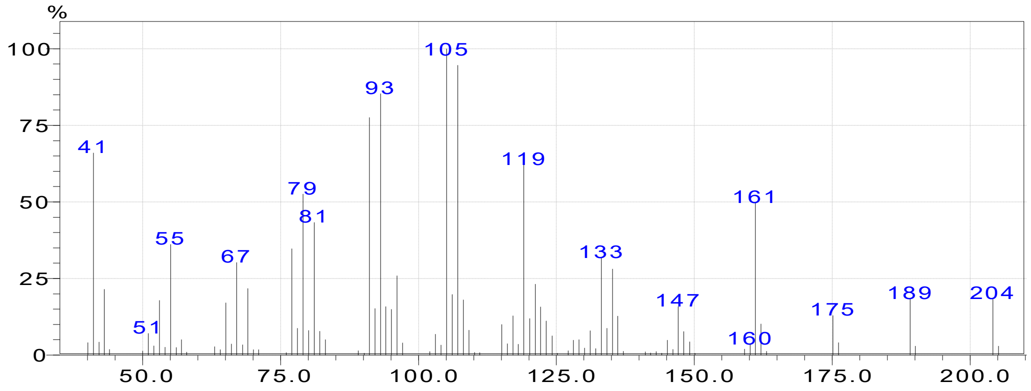

Figura 13. Mass spectrum of Viridiflorene

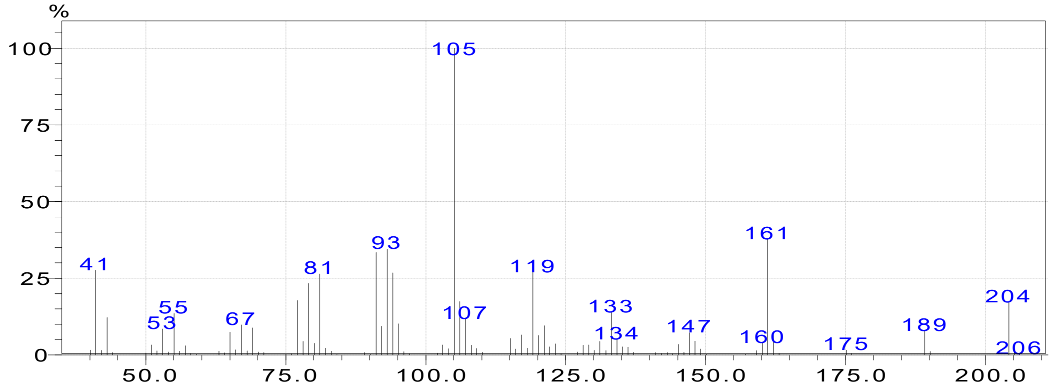

Figura 14. Mass spectrum of Muurolene<alpha->

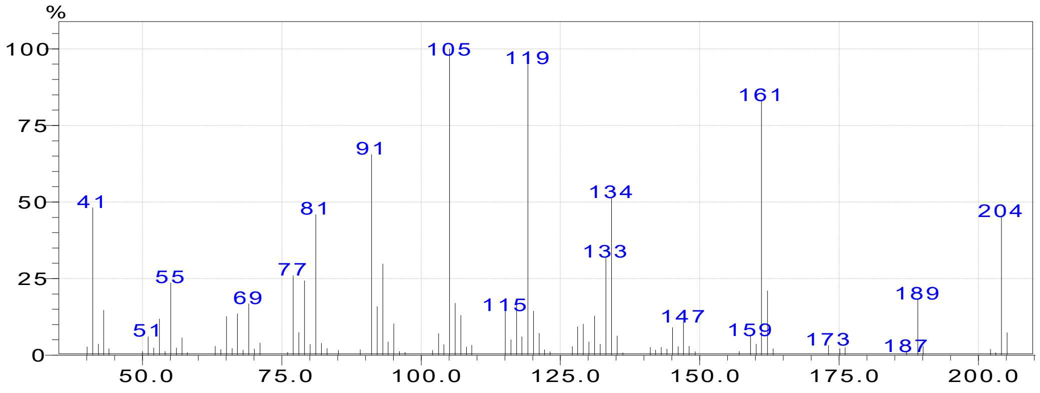

Figura 15. Mass spectrum of Amorphene <delta->

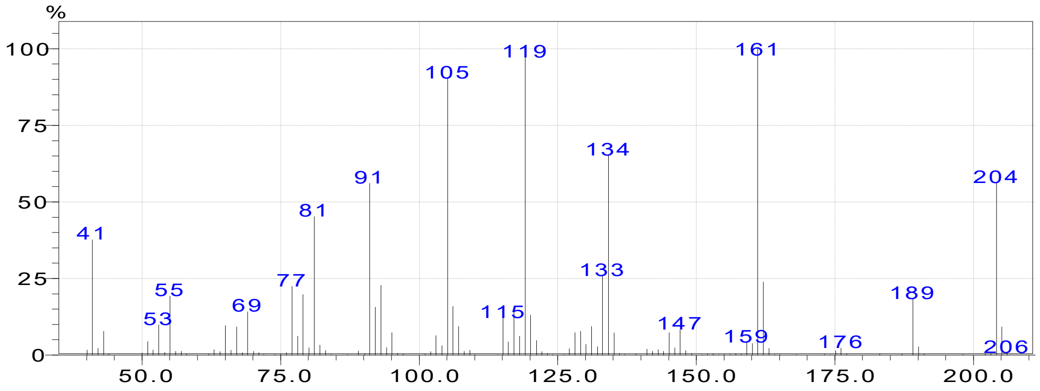

Figura 16. Mass spectrum of Cadinene<delta->

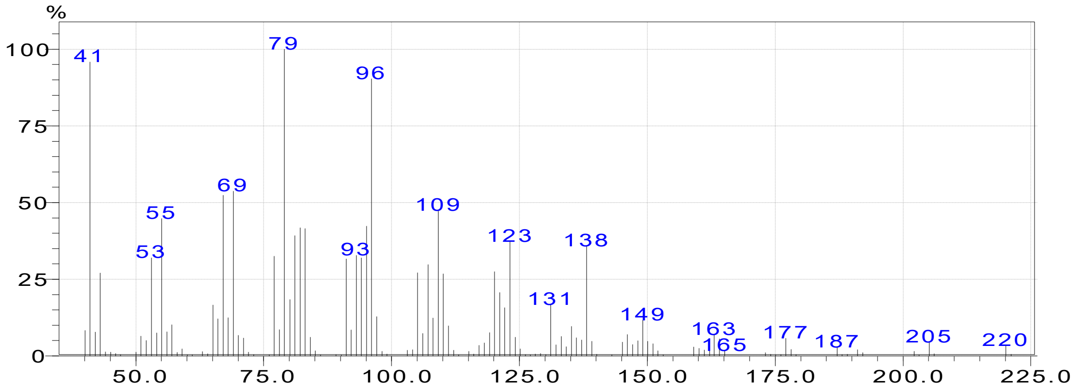

Figura 17. Mass spectrum of Italicene epoxide

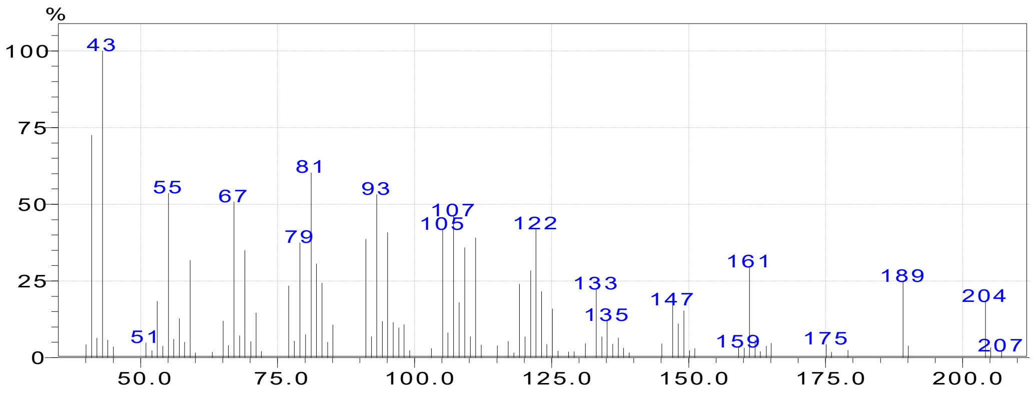

Figura 18. Mass spectrum of Palustrol

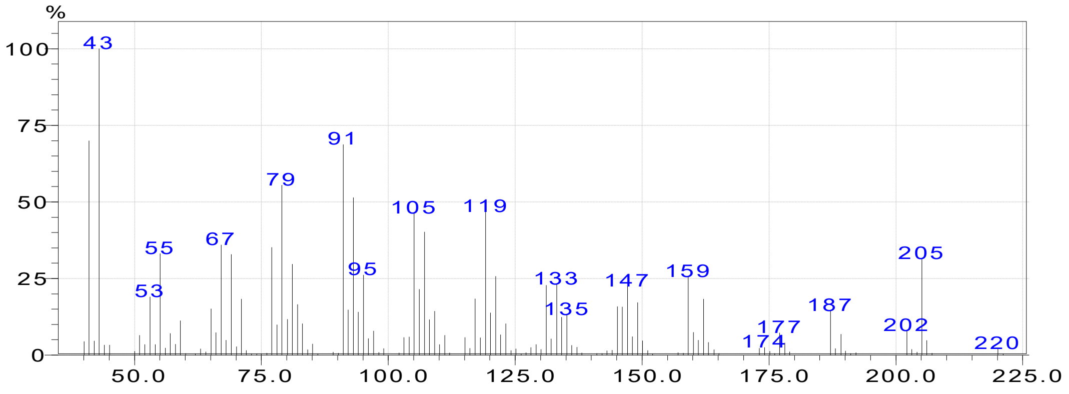

Figura 19. Mass spectrum of Spathulenol

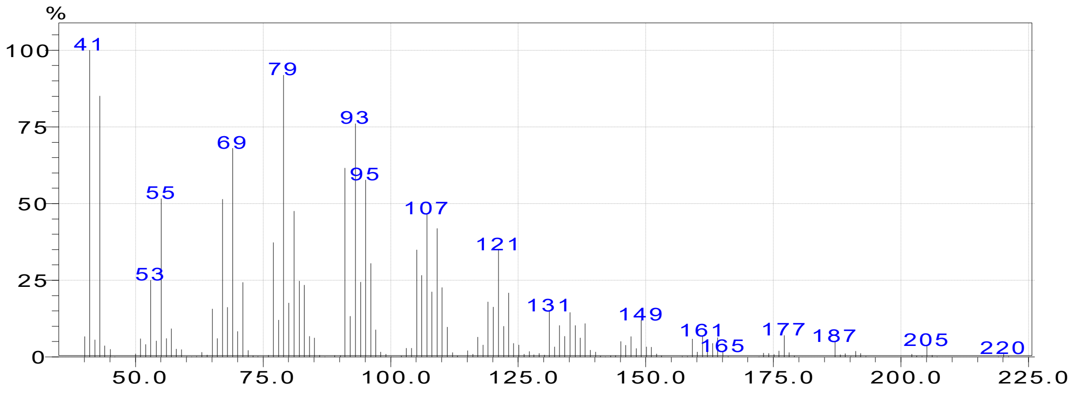

Figura 20. Mass spectrum of *Caryophyllene oxide*

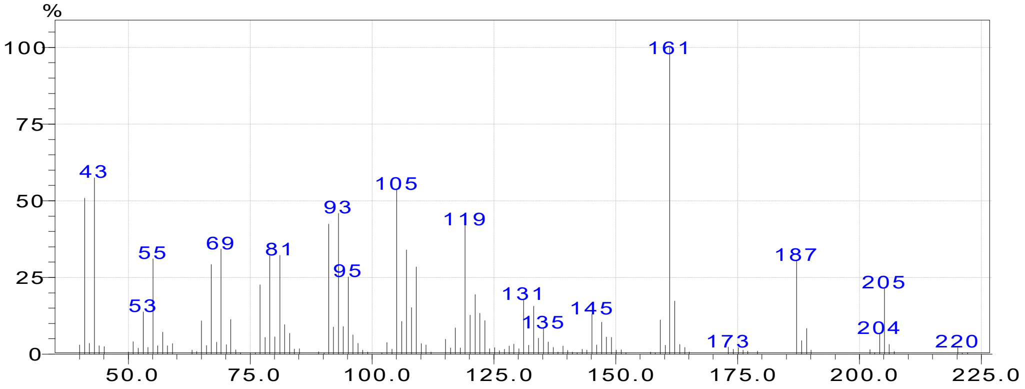

Figura 21. Mass spectrum of Viridiflorol

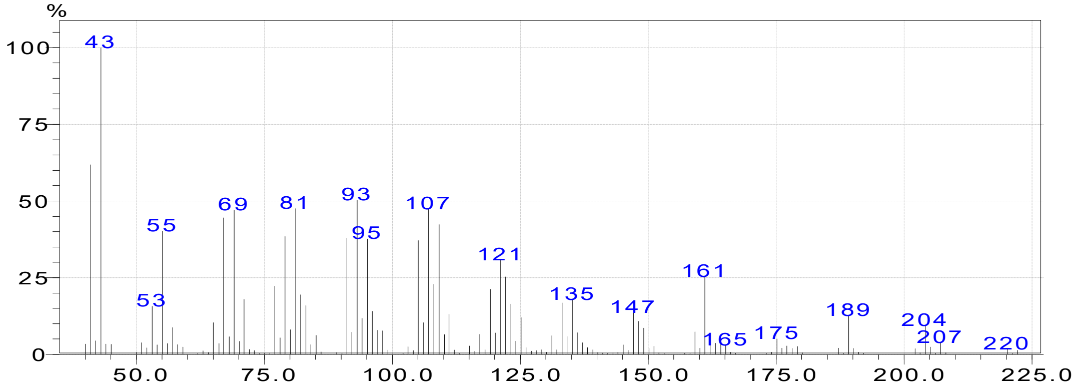

Figura 22. Mass spectrum of Globulol

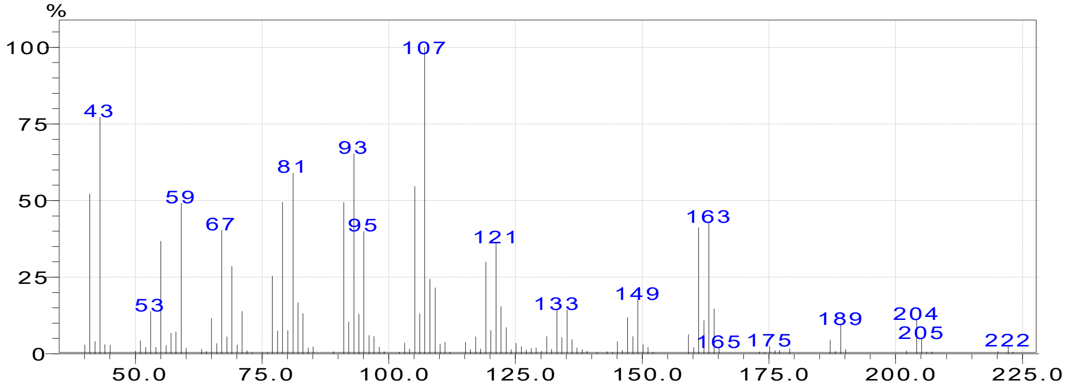

Figura 23. Mass spectrum of Cubeban-11-ol

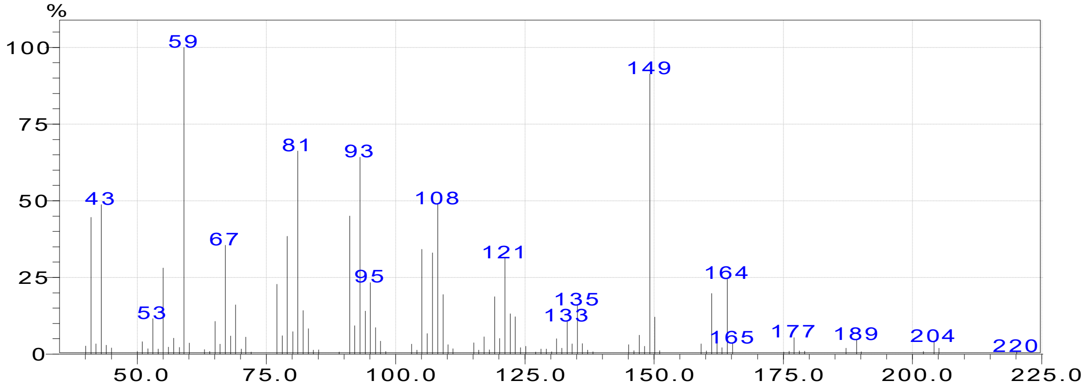

Figura 24. Mass spectrum of Rosifoliol

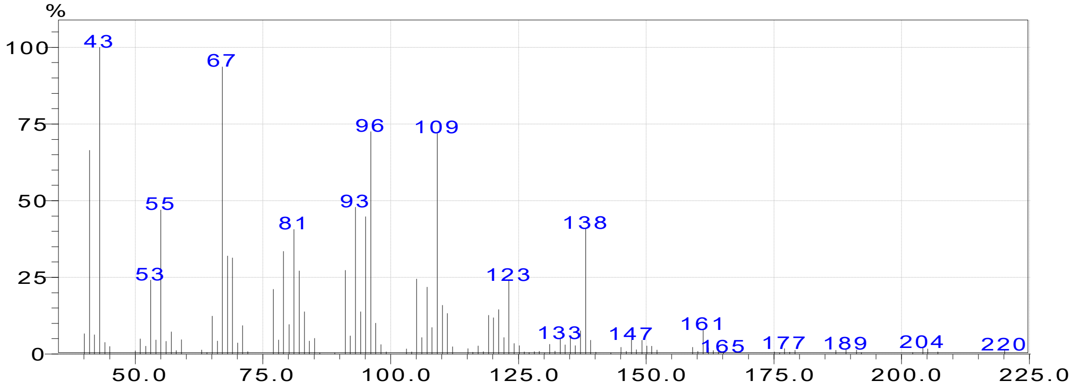

Figura 25. Mass spectrum of Humulene epoxide II

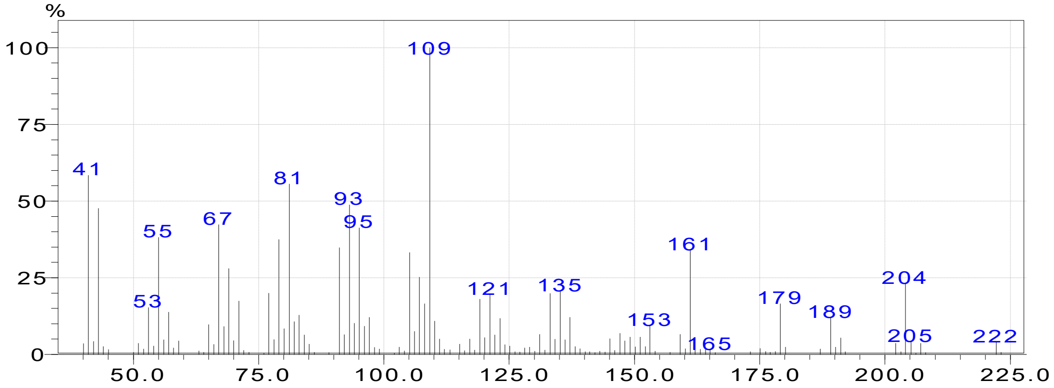

Figura 26. Mass spectrum of Junenol

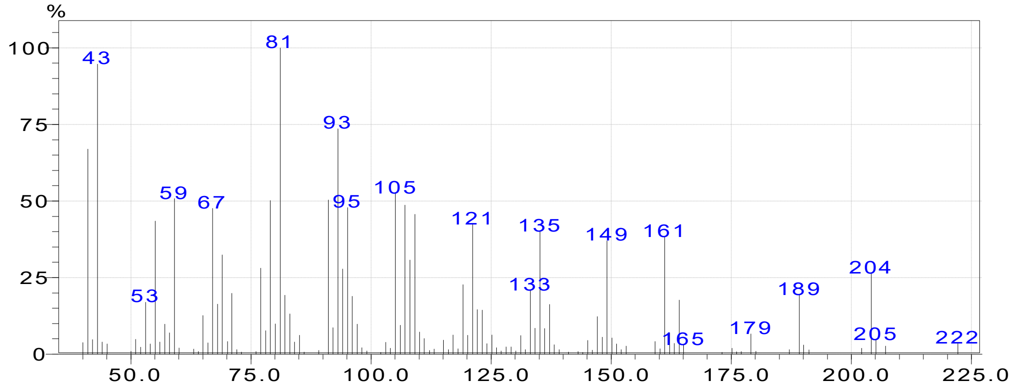

Figura 27. Mass spectrum of Eremoligenol

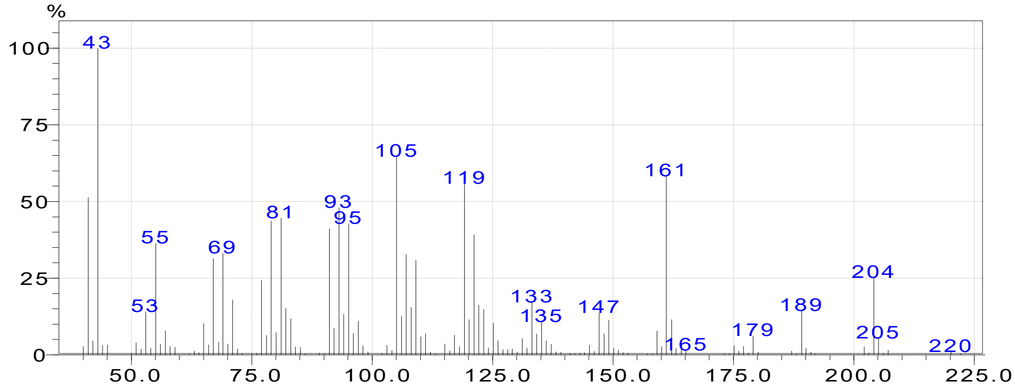

Figura 28. Mass spectrum of Cubenol<1-epi->

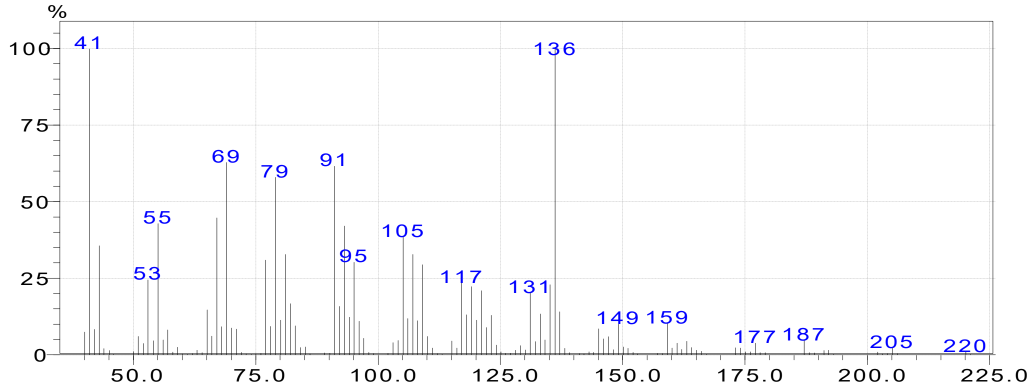

Figura 29. Mass spectrum of Caryophylla-4(12),8(13)-dien-5-alpha-ol

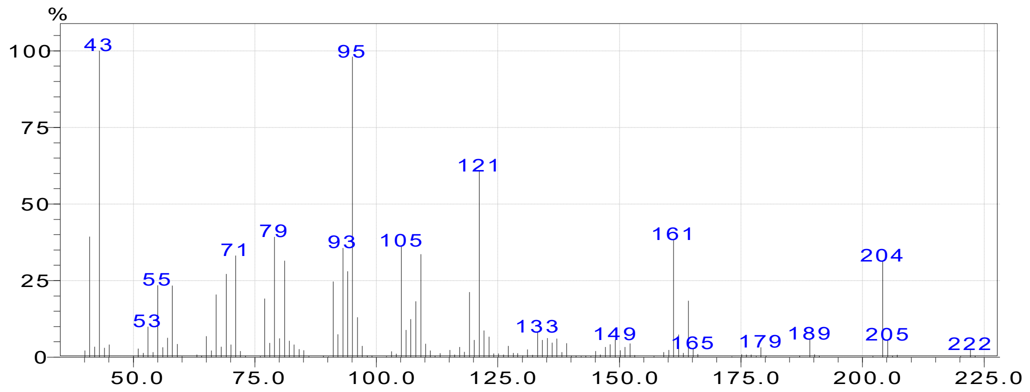

Figura 30. Mass spectrum of epi-alfa-muurolol

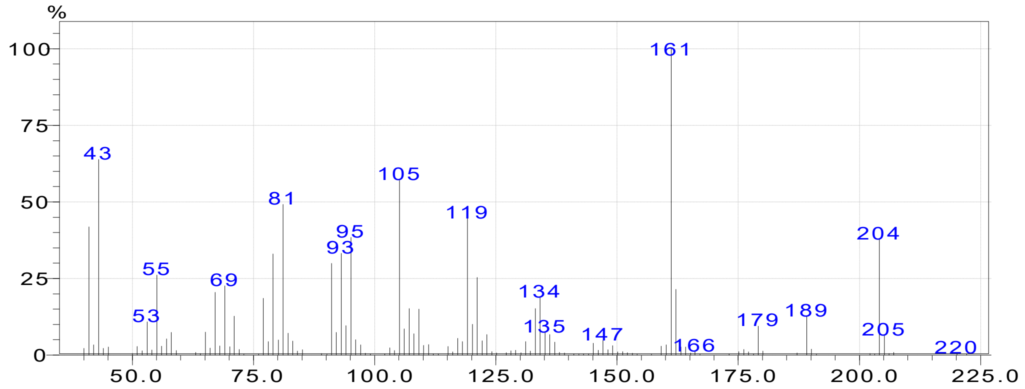

Figura 31. Mass spectrum of epi-lfa-cadinol

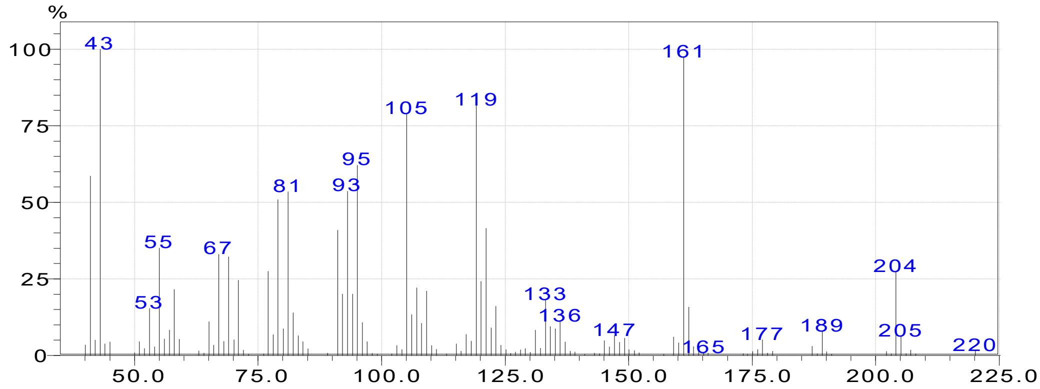

Figura 32. Mass spectrum of alfa-muurolol

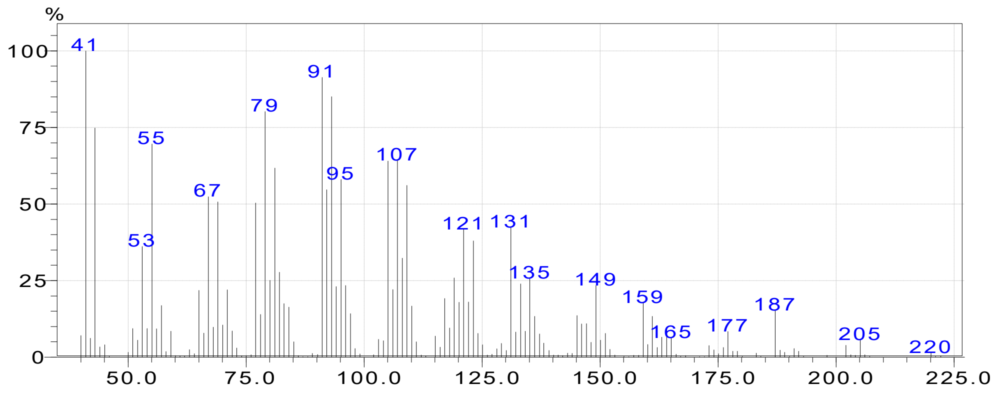

Figura 33 . Mass spectrum of Caryophyllene <14-hydroxy-9-epi-(E)->

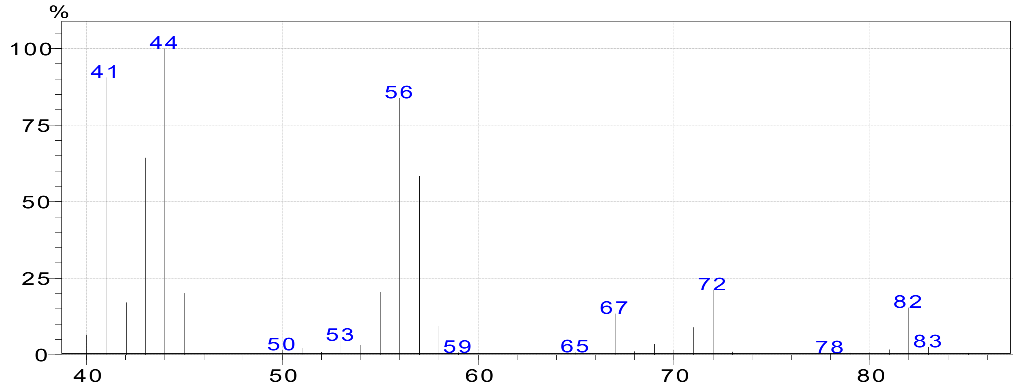

Figura 34. Mass spectrum of Hexanal

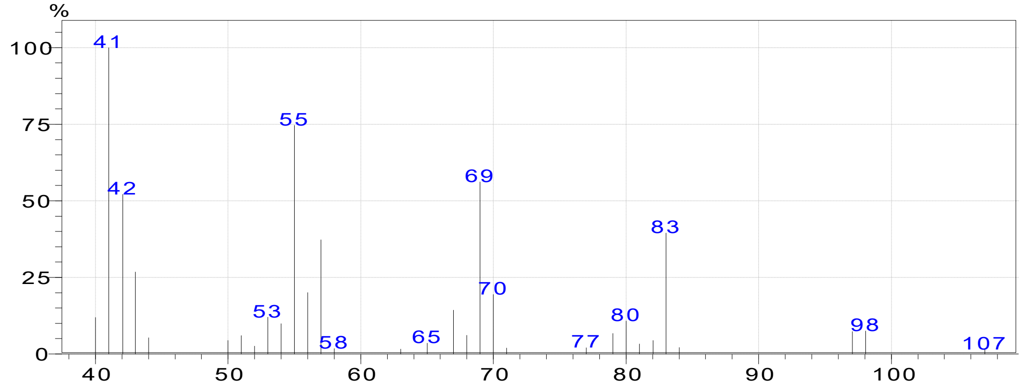

Figura 35. Mass spectrum of Hexenal<2E->

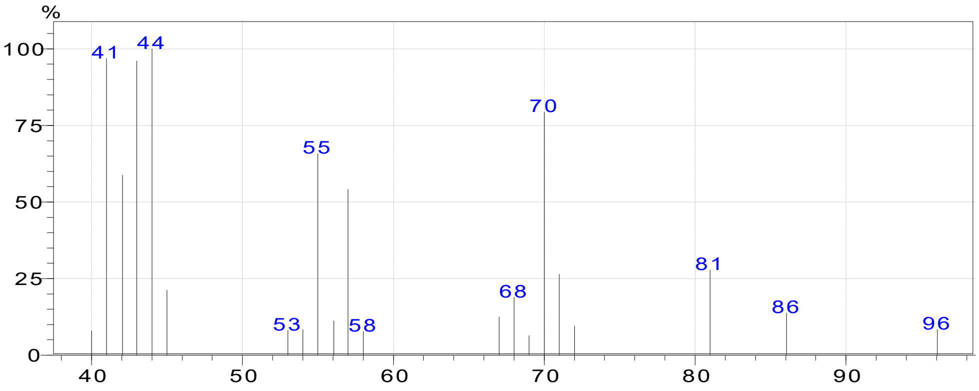

Figura 36. Mass spectrum of Heptanal

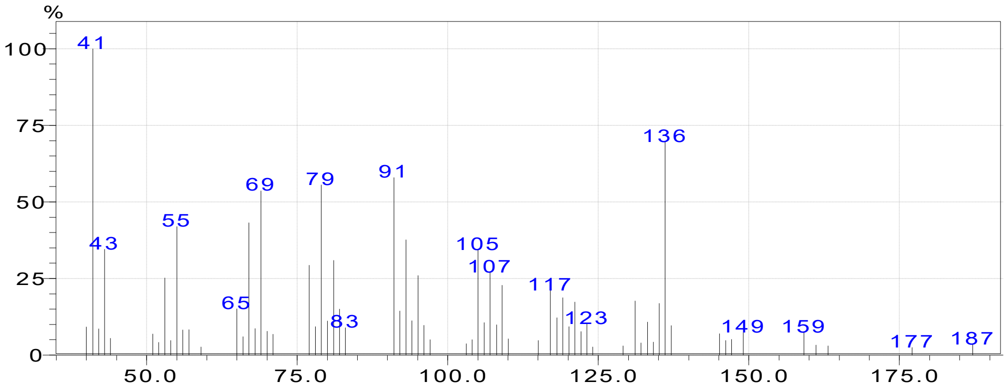

Figura 37. Mass spectrum of Caryophylla-4(12),8(13)-dien5-beta-ol

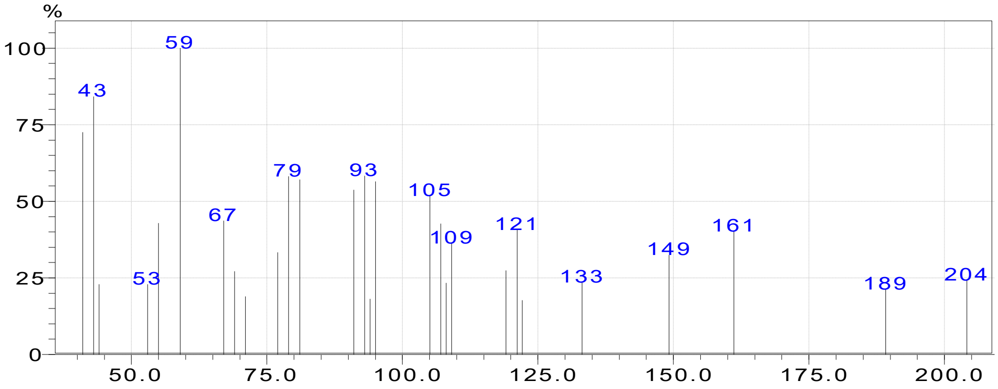

Figura 38. Mass spectrum of Himachalol

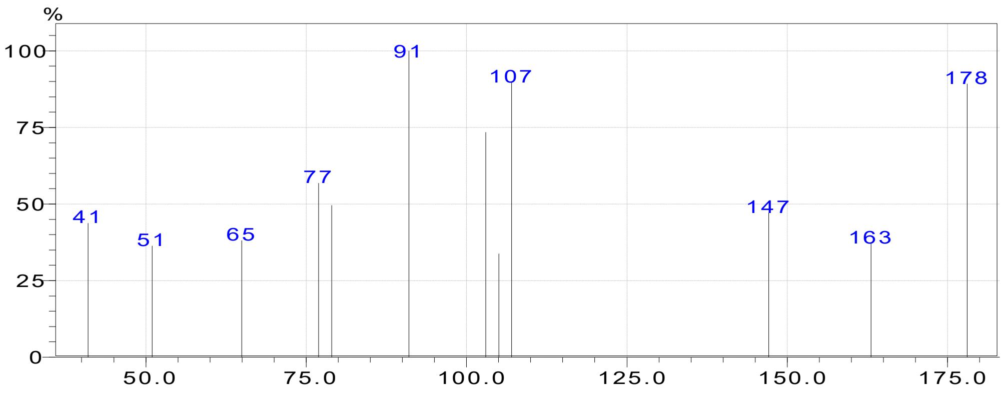

Figura 39. Mass spectrum of Eugenol <methyl->

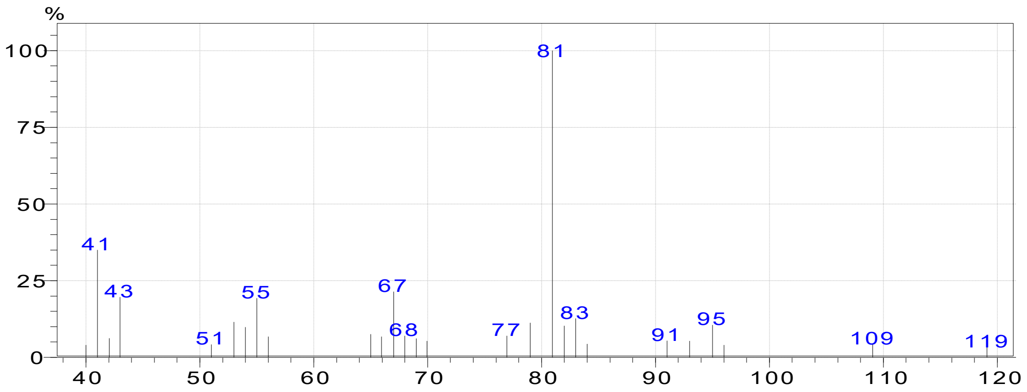

Figura 40. Mass spectrum of Undecadienal<2E,4E->

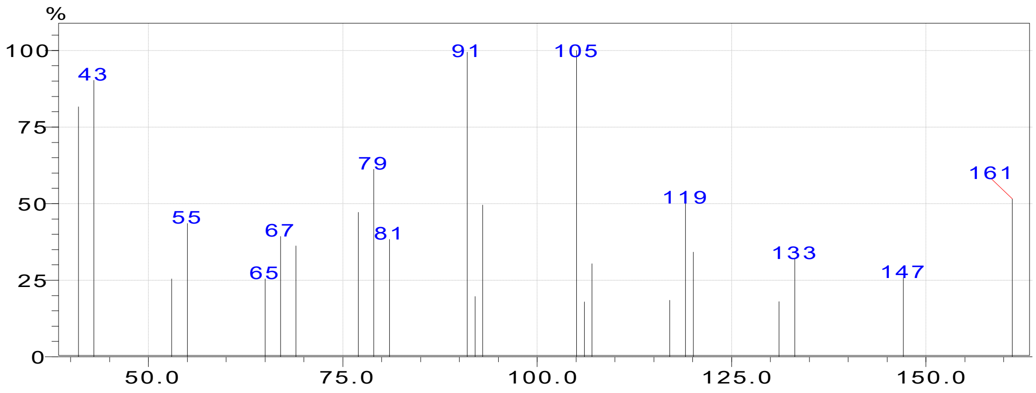

Figura 41. Mass spectrum of beta.-ylangene

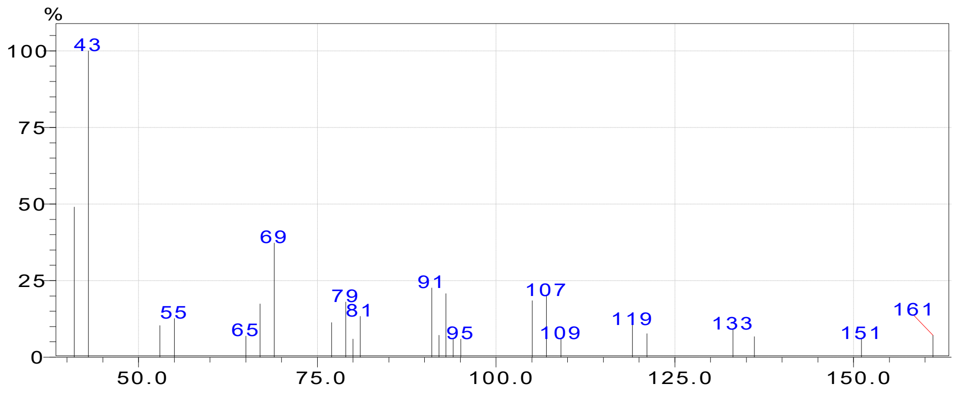

Figura 42. Mass spectrum of Geranyl acetone

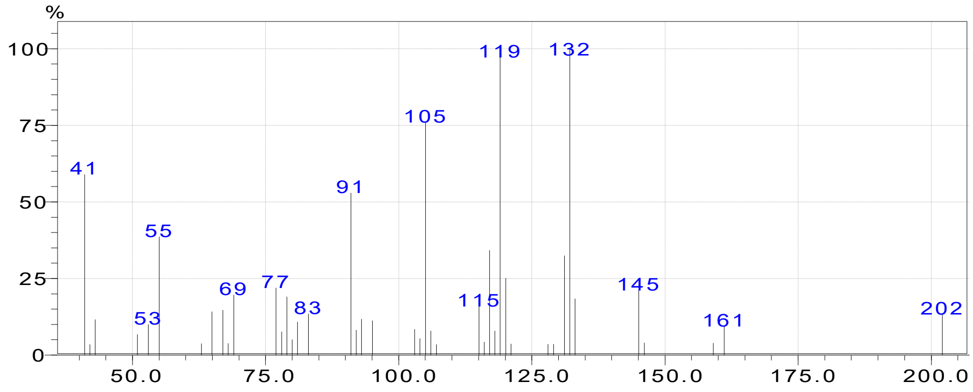

Figura 43. Mass spectrum of Curcumene<alfa->

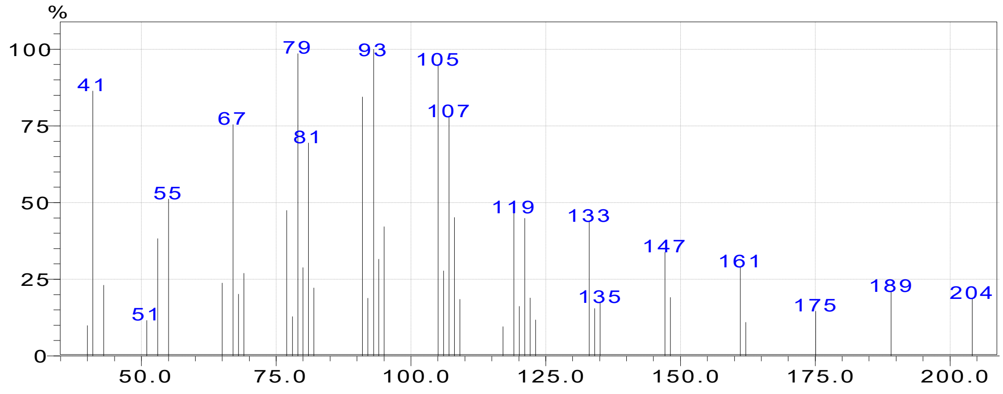

Figura 44. Mass spectrum of Selinene<beta->

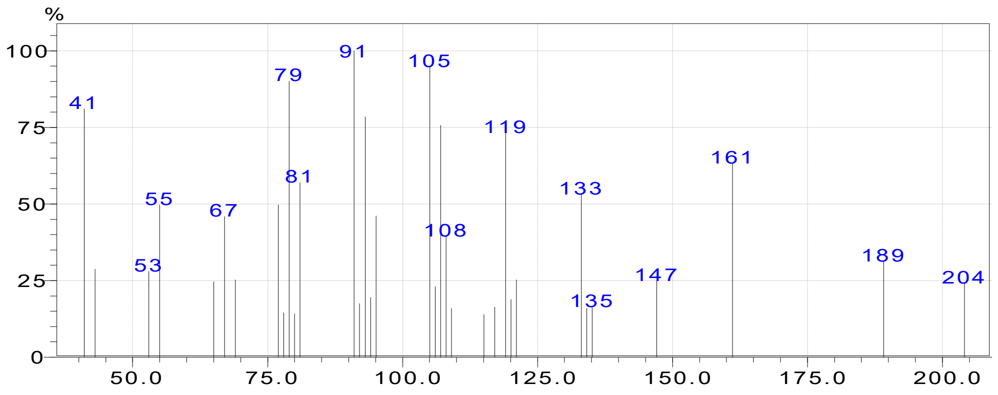

Figura 45. Mass spectrum of Valencene

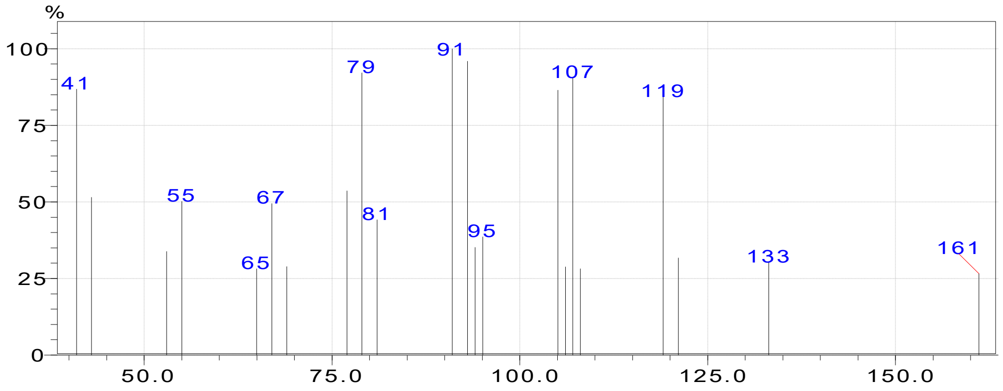

Figura *46*. Mass spectrum of Premnaspirodiene

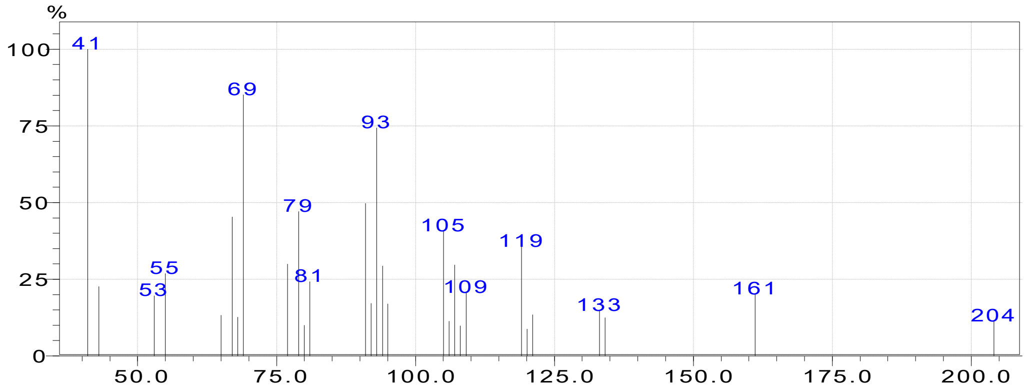

Figura 47. Mass spectrum of Bisabolene<beta->

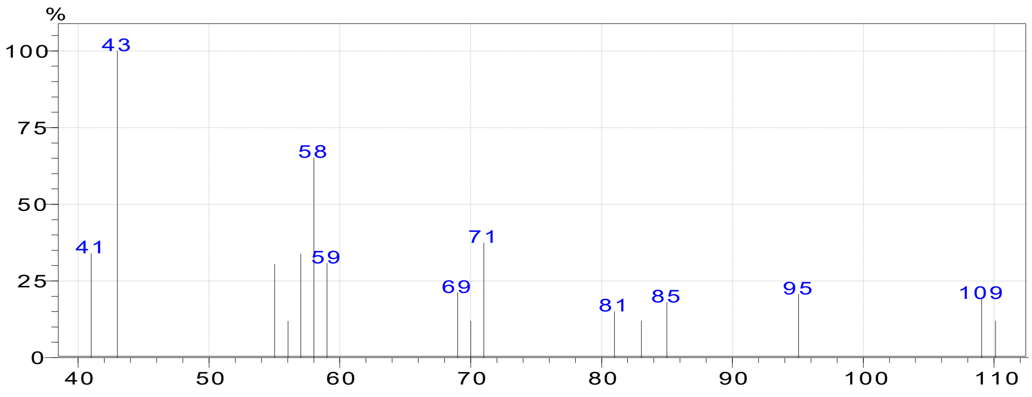

Figura 48. Mass spectrum of Phytone

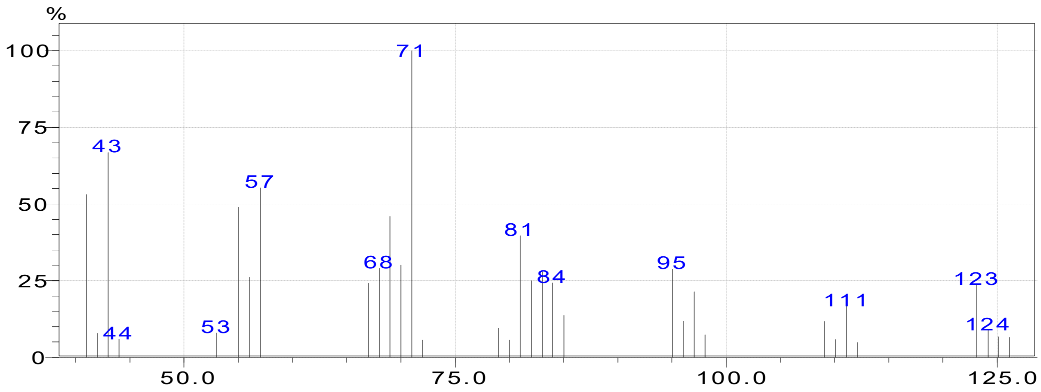

Figura 49. Mass spectrum of Phytol
